# The Trial-Ready Cohort for Preclinical/Prodromal Alzheimer’s Disease (TRC-PAD) Project: An Overview

**DOI:** 10.14283/jpad.2020.45

**Published:** 2020

**Authors:** P.S. Aisen, R.A. Sperling, J. Cummings, M.C. Donohue, O. Langford, G.A. Jimenez-Maggiora, R.A. Rissman, M.S. Rafii, S. Walter, T. Clanton, R. Raman

**Affiliations:** 1. Alzheimer’s Therapeutic Research Institute, University of Southern California, San Diego, CA, USA; 2. Center for Alzheimer Research and Treatment, Brigham and Women’s Hospital, Harvard Medical School, Boston, MA, USA; 3. Department of Brain Health, School of Integrated Health Sciences, University of Las Vegas, Nevada; Cleveland Clinic Lou Ruvo Center for Brain Health, USA; 4. Department of Neurosciences, University of California San Diego, San Diego, CA, USA

**Keywords:** Trial-Ready Cohort, Alzheimer’s disease

## Abstract

The Trial-Ready Cohort for Preclinical/prodromal Alzheimer’s Disease (TRC-PAD) project is a collaborative effort to establish an efficient mechanism for recruiting participants into very early stage Alzheimer’s disease trials. Clinically normal and mildly symptomatic individuals are followed longitudinally in a web-based component called the Alzheimer’s Prevention Trial Webstudy (APT Webstudy), with quarterly assessment of cognition and subjective concerns. The Webstudy data is used to predict the likelihood of brain amyloid elevation; individuals at relatively high risk are invited for in-person assessment in the TRC screeing phase, during which a cognitive battery is administered and Apolipoprotein E genotype is obtained followed by reassessment of risk of amyloid elevation. After an initial validation study, plasma amyloid peptide ratios will be included in this risk assessment. Based on this second risk calculation, individuals may have amyloid testing by PET scan or lumbar puncture, with those potentially eligible for trials followed in the TRC, while the rest are invited to remain in the APT Webstudy. To date, over 30,000 individuals have participated in the Webstudy; enrollment in the TRC is in its early stage.

## Introduction

The critical need for effective disease-slowing therapy for Alzheimer’s disease (AD) is among the most important health care challenges. Advances in understanding the biology of AD reveal that the disease has a 15-20 year preclinical period during which individuals are cognitively normal but have fibrillar brain amyloid, a prodromal phase during which mild cognitive impairment is present, and a dementia phase with more severe cognitive and functional compromise (1). Disease-modifying therapies may best be evaluated in the early stages of AD when it seems most feasible to preserve cognition and forestall decline. Amyloid changes are the earliest identifiable biological changes of AD, and recent trials of anti-amyloid agents in patients with prodromal AD and very mild AD dementia suggest that anti-amyloid approaches may be viable therapeutics in early stages of the illness. Aducanumab, BAN2401 and gantenerumab have all had outcomes in recent trials suggesting clinical or biological benefit in symptomatic participants [https://investors.biogen.com/news-releases/news-release-details/biogen-plans-regulatory-filing-aducanumab-alzheimers-disease]; suggesting that that earlier treatment, before the extensive accumulation of amyloid plaques and irreversible synaptic damage, may provide clinically meaningful gains. Indeed, it may be the case that all disease-modifying strategies may need to target individuals that are at early, preclinical points on the Alzheimer’s continuum (2).

Very early, large, intervention trials are feasible. The A4 (Anti-Amyloid treatment in Asymptomatic Alzheimer’s) trial, for example, is a multicenter trial in sporadic pre-symptomatic AD that demonstrated that clinically normal individuals 65 years of age and older can be screened for amyloid elevation using positron emission tomography (PET) and enrolled in a longterm, placebo-controlled treatment study (3). A total of 1169 individuals were randomized into A4, though the recruitment process took over three years.

Recruitment challenges are especially severe for trials in preclinical and prodromal AD populations, in which the minimal nature or absence of cognitive symptoms means that individuals do not to seek medical care for memory decline. While AD dementia trials typically recruit from medical practices and clinics specializing in caring for patients with cognitive disorders, the preclinical AD population requires a different approach. Clinically normal A4 participants were identified by screening on the basis of age alone. As expected, about 30% of asymptomatic individuals 65 years or older were amyloid positive by PET.

Therefore a large number of cognitively normal individuals needed to be screened with a lengthy and expensive process (including education, behavioral assessment prior to scanning, then scanning and disclosure) in order to fully enroll this prevention trial. Thus, early stage trials require a method to efficiently connect with individuals who are concerned about their risk for AD and pre-screening to identify those individuals who are at high-risk in order to reduce the high costs and delay associated with a high screen-fail rate (4).

The Trial-Ready Cohort in Preclinical/prodromal Alzheimer’s disease (TRC-PAD) program grew out of a series of meetings of academic and industry investigators, organized by the Global Alzheimer Platform (GAP) to address the challenges of early stage trial recruitment (5). An academic team filed a successful application to the National Institute on Aging, and the program was launched in early 2018. This overview summarizes the considerations behind the design and implementation of TRC-PAD.

## Overall design elements and the APT Webstudy

The TRC-PAD project aims to establish a recruitment infrastructure for early stage AD trials that will shorten the enrollment period from years to months. Participants are drawn from existing registries (“feeders”) plus media and outreach efforts to join the Alzheimer Prevention Trials Webstudy (APT Webstudy). The Webstudy is an online tool designed to collect brief information on demographics, family history, medical history, and subjective cognitive concerns. Unsupervised cognitive assessment collects data on intellectual and memory function relevant to possible early AD. Participants are asked to return to the site quarterly to provide longitudinal cognitive and subjective data. Each participant’s demographic and cognitive data inform his/her individualized risk assessment. The APT Webstudy data is analyzed in an adaptive algorithm using statistical models to determine likelihood of elevation in brain amyloid; initial algorithms are based primarily on analysis of the pre-randomization data from the A4 trial (10). Based on the risk determination, as well as proximity to active TRC-PAD clinical sites and the entry criteria for available trials, individuals may be invited for in-person assessment (including Preclinical Alzheimer Cognitive Composite (PACC) testing and apolipoprotein E (APOE) genotyping) and, based on the updated risk assessment, amyloid testing by amyloid PET or lumbar puncture for measurement of cerebrospinal fluid (CSF) amyloid peptides. Those with amyloid results consistent with AD are invited to be cohort participants, followed in-person longitudinally and ready for enrollment into trials. Those without amyloid abnormalities continue to be followed remotely in the APT Webstudy to continue to provide data for updated risk assessments.

The demographic characteristics of individuals currently enrolled in the APT Webstudy are provided in the companion paper in this issue (11).

## Building on existing registries

In addition to common strategies such as earned media coverage and social media advertising, we sought to build on prior efforts to connect with the concerned, aging population through registries. Examples of such registries are the Brain Health Registry (BHR) (12), the Alzheimer’s Prevention Registry (APR) (13) and the Alzheimer’s Association TrialMatch program (https://www.alz.org/alzheimers-dementia/research_progress/clinical-trials/about-clinical-trials). We partnered with investigators from these efforts to inform and invite registrants to the APT Webstudy. The APR, with 75,000 registrants agreeing to be contacted by researchers, was particularly successful in generating Webstudy participants.

While we have exceeded our anticipated rate of accrual with 30,000 consented participants to the APT Webstudy, and a rate of 1,000 participants consenting every month in the past year, we have not been successful in attracting an inclusive group of participants representative of the U.S. population (14). The priority of this next phase of the program will be to address this deficiency through recruitment in Spanish language, and other community-based approaches.

## Designing a low-burden, informative longitudinal study to assess risk

A challenge noted by registries in the field is that participant retention can be low. In the APT Webstudy, we can estimate risk of amyloid elevation using cross-sectional data from the pre-randomization phase of A4, but longitudinal change in subjective concerns and cognitive performance are expected to significantly improve accuracy. We have tried to improve retention by minimizing participant burden, keeping follow-up visits to 20 minutes or less, and by optimizing engagement, through sharing of graphical representations of longitudinal performance as well as up-to-date information on available and expected therapeutic trials using the Webstudy itself as well as quarterly newsletters. We provide timely responses, by email or phone, to all queries from participants. These efforts are ongoing; more work toward this goal is required.

## SRS: a data system to connect high-risk Webstudy participants to TRC sites for in-person testing

Webstudy participants determined to have relatively high risk for amyloid elevation in brain and are located near a TRC-PAD clinical site are invited to have in-person assessments to screen for enrollment into the TRC. In addition to predicted amyloid PET SUVr levels, the selection process considers demographics to achieve diversity, particularly important since Webstudy participants tend to be homogeneous. At this time, the final selections are manually reviewed; after gaining more experience with the system, we will increase automation. Selected participants are presented to site teams through the Site Referral System (SRS) described in a later paper in this series (15). In instances where participants in the APT Webstudy are do not reside close to a TRC-PAD site, they are provided with the opportunity to download and print a report that displays their performance on the various assessments as well as an explanation of the assessments, that they can review with their healthcare provider.

TRC sites are provided a list of potential participants on a monthly basis; the size of the geographic referral area and the number of participants to be referred customized based on site capacity and recruitment needs.

## Recalculation of risk and assessment of potential trial eligibility to select for amyloid imaging

Amyloid testing, by PET or CSF analysis, is an expensive and somewhat invasive component of the assessment of early stage trial eligibility. TRC-PAD aims to dramatically reduce the number of amyloid tests required to recruit trial participants. The first in-person visit of participants referred via the SRS to TRC sites includes confirmation of demographic information, medical and neurological assessment, cognitive testing with the PACC and APOE genotyping; these data allow a more precise prediction of brain amyloid level. APOE genotype in particular substantially improves prediction of brain amyloid; if APOE genotype is included in the risk assessment, almost all selected would be APOE ε4 carriers. Our target trial sample will have a distribution of APOE genotypes representative of the AD population, meaning 30-40% APOE ε4 non-carriers. We therefore assess risk separately for carriers and non-carriers to allow control over final genetic distribution. Again, the selection process for amyloid testing permits adjustment to support diversity goals.

## Enrollment in TRC based on SUVr or CSF amyloid peptide ratio

Eligibility criteria for the TRC is based on criteria, including amyloid levels, for preclinical and prodromal clinical trials anticipated to be available at each site. The AHEAD 3-45 platform, a public private partnership collaboration of the NIA Alzheimer’s Clinical Trials Consortium and Eisai Pharmaceuticals including most TRC sites, is currently in its start-up phase; this program will enroll clinically normal individuals with elevated and intermediate levels of amyloid. Current TRC amyloid requirements are based on the this platform. Amyloid-eligible individuals are invited to join the TRC for semiannual in-person reassessment including PACC testing.

## Connection to early-stage clinical trials

The informatics architecture for TRC-PAD envisions use of longitudinal TRC data as run-in data for therapeutic trials. The system is seamlessly integrated with the Alzheimer’s Treatment Research Institute/Alzheimer’s Clinical Trial Consortium (ATRI/ACTC) Electronic Data System (EDC), is 21 CFR Part 11 compliant, and supports the inclusion of TRC data in trial datsets.

Selection of TRC participants to specific trials available at a site is based on the preferences of participants in discussions with their site investigators. While TRC-PAD procedures are designed with ongoing or coming ATRI/ACTC trials in mind, participants may choose to be screened for any available trials. Additionally, the TRC is designed to allow participants to return after beling either screened or participating in a clinical trial, meeting the important need to the field of retaining and following screen fails.

## Incorporation of plasma abeta ratios into the TRC-PAD amyloid risk assessment

In a newly funded revision of the TRC-PAD program, we are now in the process of integrating plasma amyloid peptide ratio assays into the final risk assessment in-person screening prior to brain amyloid testing. The promise of plasma amyloid ratio testing has been confirmed by two independent labs using different immunoprecipitation/mass spectrometry approaches (16, 17); each finds a strong association between plasma ratios and brain amyloid load. Encouraging results have also been reported using an automated immunoassay (18). We will assess these methods by obtaining plasma prior to brain amyloid testing for the initial few hundred APT Webstudy participants to undergo brain amyloid PET. The optimal pre-processing approach and assay methodology will then be incorporated into the risk algorithm for the remaining participants. We expect a substantial improvement in accuracy of our algorithm, as well as a significant reduction the number of negative amyloid PET scans and CSF draws, reducing burden to participants and high cost of screening.

## TRC-PAD and Primary Prevention of AD

Our ultimate goal is the primary prevention of AD. This will require monitoring individuals prior to amyloid elevation in brain to identify characteristics (demographic, genetic, biochemical, clinical) that predict later amyloid elevation, enabling the selection of high-risk people for primary prevention trials involving reducing production or promoting clearance of amyloid peptides. We believe that the APT Webstudy, with the addition of remote acquisition of DNA, and longitudinal collection of blood to assess Aβ42/Aβ40 ratios over time (5), will provide the necessary infrastructure for this effort. Plasma assays of Aβ42/Aβ40 followed longitudinally will be key; encouraging data suggest that plasma amyloid ratios predict later amyloid PET positivity (16).

The TRC-PAD program is a work in progress. While we have passed our initial target of 25,000 registrants in the APT Webstudy, TRC screening and amyloid testing are still in a very early stage, and validation of a plasma abeta ratio assay is still in the future. Many investigators across the U.S. and around the world are contributing to the continued optimization and implementation of TRC-PAD. We hope that this program will accelerate recruitment into early intervention AD trials and facilitate work toward the primary prevention of AD.

## Figures and Tables

**Figure 1.**
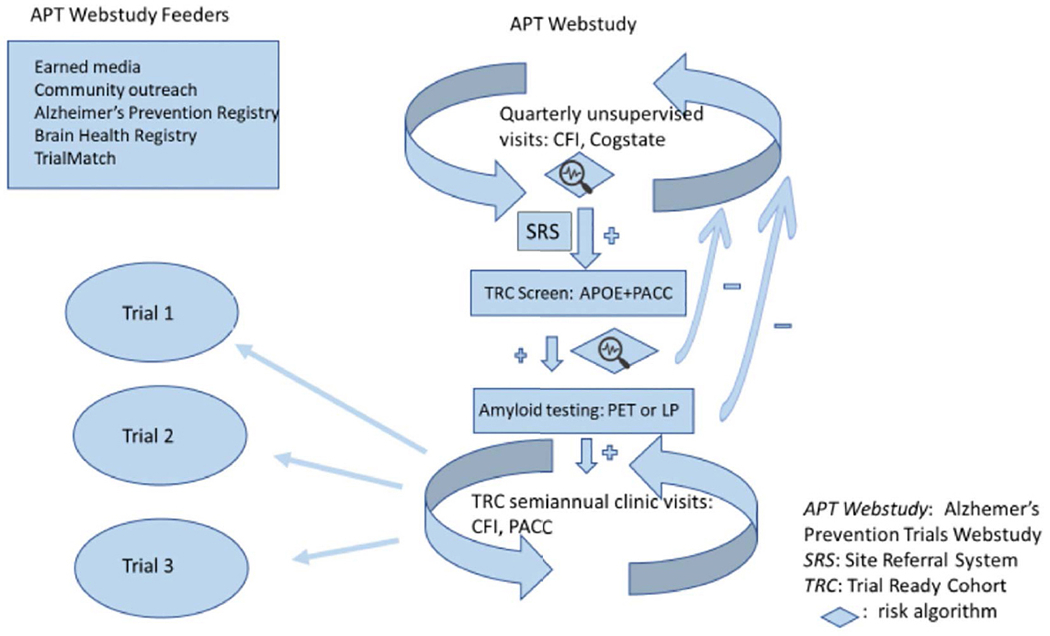
The TRC-PAD program. APOE - apolipoprotein E genotype; CFI – Cognitive Function Instrument (6, 7); LP – lumbar puncture; Cogstate – Cogstate Brief Battery (8); PACC - Preclinical Alzheimer Cognitive Composite (9)

## Key Elements of the TRC-PAD Program

APT Webstudy: online unsupervised longitudinal data collection, including cognitive performance and subjective concerns, to inform initial assessment of risk of amyloid elevation. Participants are invited to visit TRC-PAD sites based on this risk determination, along with geographic and demographic factors

Site Referral System: the informatics module connecting the selected APT Webstudy participants to the sites for in-person screening and refinement of risk assessment

Amyloid testing: Amyloid PET scan or cerebrospinal fluid amyloid peptide assays determine eligibility for the Trial Ready Cohort (TRC)

TRC: Individuals are followed in-person at TRC-PAD sites until they are enrolled in a trial. TRC data are collected in a CFR Part 11 compliant system integrally linked to an Electronic Data Capture trial system so that it can be used as run-in data for therapeutic studies

## References

[1] Jack CR Jr., Albert MS, Knopman DS, Introduction to the recommendations from the National Institute on Aging-Alzheimer’s Association workgroups on diagnostic guidelines for Alzheimer’s disease. Alzheimers Dement. 2011;7(3):257–262. 21514247 10.1016/j.jalz.2011.03.004 PMC3096735

[2] Aisen PS, Cummings J, Jack CR Jr., On the path to 2025: understanding the Alzheimer’s disease continuum. Alzheimers Res Ther. 2017;9(1):60. 28793924 10.1186/s13195-017-0283-5 PMC5549378

[3] Sperling RA, Donohue MC, Raman R, Association of Factors With Elevated Amyloid Burden in Clinically Normal Older Individuals. JAMA Neurol. 2020. 10.1001/jamaneurol.2020.0387 PMC7136861 32250387

[4] Rafii MS, Aisen PS. Alzheimer’s Disease Clinical Trials: Moving Toward Successful Prevention. CNS Drugs. 2019;33(2):99–106. 30560544 10.1007/s40263-018-0598-1 PMC6367028

[5] Sperling R, Cummings J, Donohue M, Aisen P. Global Alzheimer’s Platform Trial Ready Cohorts for the Prevention of Alzheimer’s Dementia. J Prev Alzheimers Dis. 2016;3(4):185–187. 29199318 10.14283/jpad.2016.108

[6] Walsh SP, Raman R, Jones KB, Aisen PS, Alzheimer’s Disease Cooperative Study G. ADCS Prevention Instrument Project: the Mail-In Cognitive Function Screening Instrument (MCFSI). Alzheimer Dis Assoc Disord. 2006;20(4 Suppl 3):S170–178. 17135810 10.1097/01.wad.0000213879.55547.57

[7] Amariglio RE, Donohue MC, Marshall GA, Tracking early decline in cognitive function in older individuals at risk for Alzheimer disease dementia: the Alzheimer’s Disease Cooperative Study Cognitive Function Instrument. JAMA Neurol. 2015;72(4):446–454. 25706191 10.1001/jamaneurol.2014.3375 PMC4397164

[8] Maruff P, Thomas E, Cysique L, Validity of the CogState brief battery: relationship to standardized tests and sensitivity to cognitive impairment in mild traumatic brain injury, schizophrenia, and AIDS dementia complex. Arch Clin Neuropsychol. 2009;24(2):165–178. 19395350 10.1093/arclin/acp010

[9] Donohue MC, Sperling RA, Salmon DP, The preclinical Alzheimer cognitive composite: measuring amyloid-related decline. JAMA Neurol. 2014;71(8):961–970. 24886908 10.1001/jamaneurol.2014.803 PMC4439182

[10] Langford O, Raman R, Sperling RA, Predicting Amyloid Burden to Accelerate Recruitment of Secondary Prevention Clinical Trials. J Prev Alz Dis 2020;4(7):213–218 10.14283/jpad.2020.44 PMC7745538 32920622

[11] Walter S, Langford OG, Clanton TB, The Trial-Ready Cohort for Preclinical and Prodromal Alzheimer’s Disease (TRC-PAD): Experience from the First 3 Years. J Prev Alz Dis 2020;4(7):234–241 10.14283/jpad.2020.47 PMC7767585 32920625

[12] Weiner MW, Nosheny R, Camacho M, The Brain Health Registry: An internet-based platform for recruitment, assessment, and longitudinal monitoring of participants for neuroscience studies. Alzheimers Dement. 2018;14(8):1063–1076. 29754989 10.1016/j.jalz.2018.02.021 PMC6126911

[13] Langbaum JB, Karlawish J, Roberts JS, GeneMatch: A novel recruitment registry using at-home APOE genotyping to enhance referrals to Alzheimer’s prevention studies. Alzheimers Dement. 2019;15(4):515–524. 30772251 10.1016/j.jalz.2018.12.007 PMC6461487

[14] Walter S, Clanton TB, Langford OG, Recruitment into the Alzheimer Prevention Trials (APT) Webstudy for a Trial-Ready Cohort for Preclinical and Prodromal Alzheimer’s Disease (TRC-PAD). J Prev Alz Dis 2020;4(7):219–225 10.14283/jpad.2020.46 PMC7842199 32920623

[15] Jimenez-Maggiora GA , Bruschi S, Raman R, TRC-PAD: Accelerating Recruitment of AD Clinical Trials through Innovative Information Technology. J Prev Alz Dis 2020;4(7):226–233. 10.14283/jpad.2020.48 PMC7769128 32920624

[16] Schindler SE, Bollinger JG, Ovod V, High-precision plasma beta-amyloid 42/40 predicts current and future brain amyloidosis. Neurology. 2019;93(17):e1647–e1659. 31371569 10.1212/WNL.0000000000008081 PMC6946467

[17] Nakamura A, Kaneko N, Villemagne VL, High performance plasma amyloid-beta biomarkers for Alzheimer’s disease. Nature. 2018;554(7691):249–254. 29420472 10.1038/nature25456

[18] Palmqvist S, Janelidze S, Stomrud E, Performance of Fully Automated Plasma Assays as Screening Tests for Alzheimer Disease-Related beta-Amyloid Status. JAMA Neurol. 2019. 10.1001/jamaneurol.2019.1632 PMC6593637 31233127

